# A novel composite of chitosan and *Bacillus subtilis* exopolysaccharide for the removal of methylene blue from aqueous solutions

**DOI:** 10.1038/s41598-026-36875-7

**Published:** 2026-02-12

**Authors:** Mohamed Hemida Abd-Alla, Elhagag A. Hassan, Esraa A. Mohammed, Shymaa R. Bashandy

**Affiliations:** Botany and Microbiology Department, Faculty of Science, Assiut University, Assiut, 71516 Egypt

**Keywords:** *Bacillus subtilis*, Biosorption, Chitosan-exopolysaccharide composite, Langmuir and Freundlich models, Methylene blue, Chemistry, Environmental sciences, Materials science

## Abstract

Dye pollution in water poses serious health and ecological risks, requiring wastewater treatment before discharge and prompting increased research attention due to the widespread use of dyes in various industries. This study investigates the biosorption of methylene blue (MB) using a novel composite of chitosan and *Bacillus subtilis* exopolysaccharides (EPS). Fourier-transform infrared spectroscopy (FTIR) analysis confirmed the presence of essential functional groups for dye adsorption. The biosorption process was pH-dependent, with optimal removal efficiencies at pH 6 for the chitosan/EPS composite and pH 7 for chitosan alone, showing increased adsorption capacity with rising pH from 3.0 to 7.0. Contact time experiments demonstrated efficient MB removal in approximately 30 min, achieving decolorization rates of 71.6% for the composite and 60.62% for chitosan. The composite also demonstrated a higher maximum biosorption capacity (14.26 mg g^−1^) than chitosan (13.70 mg g^−1^) according to the Langmuir isotherm model, which best described the monolayer adsorption process. Kinetic analyses revealed that the pseudo-second order model best described the adsorption process, with calculated capacities closely matching experimental values (41.67 mg g^−1^ for chitosan and 45.87 mg g^−1^ for the composite). Post-adsorption FTIR results revealed involvement of –OH, –NH, –COO^−^, and –PO₄³^−^ groups via electrostatic, hydrogen-bonding, and π–π interactions, confirming the interaction between MB and functional groups on the biosorbents. These findings highlight the synergistic effect of combining chitosan with EPS, resulting in a promising, efficient, and rapidly equilibrating biosorbent for mitigating dye pollution in wastewater. This work presents the first reported use of *Bacillus subtilis* exopolysaccharide combined with chitosan as a fully biodegradable composite biosorbent, exhibiting superior adsorption capacity (14.26 mg g^−1^), markedly faster kinetics (K₂ = 0.176 g mg^−^¹ min^−^¹), and equilibrium attainment within 30 min compared to pristine chitosan and most reported biopolymer-based adsorbents. Optimizing biosorption conditions can enhance dye removal efficiencies and contribute to sustainable environmental management practices.

## Introduction

Globally, over 80% of wastewater is released into the environment without adequate treatment, significantly affecting freshwater resources^1^. Effective treatment methods are essential for addressing water scarcity by treating both municipal and industrial wastewater^2^. Dyes, widely used in industries like textiles, leather, paper, and cosmetics, are a major environmental pollutant, with global production estimated at 700,000 to 1,000,000 tons annually^3–5^. Discharging dye effluents into the environment poses aesthetic, ecological, and health risks due to their toxicity, which can lead to serious health issues such as cancer and allergies^6–8^. Methylene Blue (MB), a commonly used dye with complex aromatic structures, presents challenges in biodegradation. It is crucial to treat dye-contaminated effluents before discharge to prevent adverse environmental impacts^9^.

Recently, a variety of physical and chemical techniques have been employed for the treatment of wastewater containing dyes., each with its own advantages and limitations^10^. Chemical oxidation techniques, such as photo-assisted degradation, are effective but can be costly and energy-intensive, facing challenges with impurities like suspended solids and dissolved organic matter^11^. Physical adsorption is a quick and efficient pre-treatment method for dye removal; however, the costs associated with adsorbents, which may include hazardous materials such as hydrogel, resin, and biochars, can be significant^12–14^. While both chemical and physical methods can be successful, there is still room for improvement.

Biodegradation is a promising and cost-effective method for treating contaminants, but it can be unstable and time-consuming, often requiring combination with other methods for practical application^15^. Various treatment methods, such as ion exchange, coagulation, flocculation, membrane separation, and photocatalytic degradation, have been devised to tackle dye and pollutant removal from polluted surroundings^16^. Among these methods, adsorption is highlighted for its high efficiency and environmental friendliness, despite concerns about operational costs and losses during the regeneration of traditional adsorbents like activated carbon^17,18^. Biosorption, a cost-effective method utilizing biological materials such as microorganisms as adsorbents, enhances biodegradation processes in treating dye-contaminated wastewater. This approach involves capturing large organic molecules like dyes, followed by their decomposition^19^.

Developing efficient, safe, and renewable adsorbents is crucial for improving wastewater treatment, particularly in addressing dye pollution. Exopolysaccharides (EPS), high-molecular-weight polymers produced by microorganisms, have emerged as promising adsorbents due to their biodegradability, safety, high adsorption efficiency, and reusability^20^. Utilizing EPS for dye adsorption promotes sustainable and eco-friendly practices, making significant strides towards cleaner production methods. Research has primarily focused on enhancing EPS adsorption capabilities, optimizing conditions, and investigating underlying processes^1^.

Chitosan, a renewable, eco-friendly, and cost-effective complexing agent, is known for its non-toxic, biodegradable, and hydrophilic properties. Its polyelectrolyte behavior and high reactivity at acidic pH are attributed to its reactive hydroxyl and amine groups^21^. These characteristics make it ideal for tailored chemical modifications, particularly in wastewater treatment applications^22^. Chitosan has demonstrated efficient binding of pollutants at trace levels in both concentrated and diluted solutions. Being the only natural cationic polymer, it exhibits polycationic behavior at pH levels below 6.3, allowing for versatile applications such as beads, films, and gels^21,23^. It can be utilized in solid form for filtration or adsorption purposes, or in liquid form for coagulation, flocculation, and polymer-assisted ultrafiltration processes^24^. Cross-linked chitosan hydrogels are widely used in biosorption applications to efficiently remove dyes from wastewater, showcasing chitosan’s versatility in sustainable environmental management^24,25^. Although both materials have been individually investigated for dye biosorption, no study to date has explored their deliberate combination into a single composite to exploit synergistic interactions between the protonated amino groups of chitosan and the diverse anionic moieties (carboxyl, phosphate) of bacterial EPS. This study bridges this critical knowledge gap by developing a novel, fully biodegradable composite from chitosan and exopolysaccharide produced by *Bacillus subtilis* EA5 (GenBank OR501464). The synergistic integration is expected to dramatically increase active site density, enhance electrostatic attraction, and accelerate adsorption kinetics compared to pristine chitosan. Moreover, this approach valorizes EPS—a fermentation by-product typically discarded as waste thereby adding economic and circular-economy value while reducing reliance on petrochemical-derived adsorbents. The study aims to synthesize and characterize a novel composite material from chitosan and bacterial EPS for efficient methylene blue biosorption, optimize biosorption parameters (pH, contact time, initial dye concentration), and investigate the adsorption kinetics and isotherms of methylene blue onto chitosan/EPS composites.

## Results and discussion

### FTIR analysis of the prepared biosorbents

**Fig. 1:**
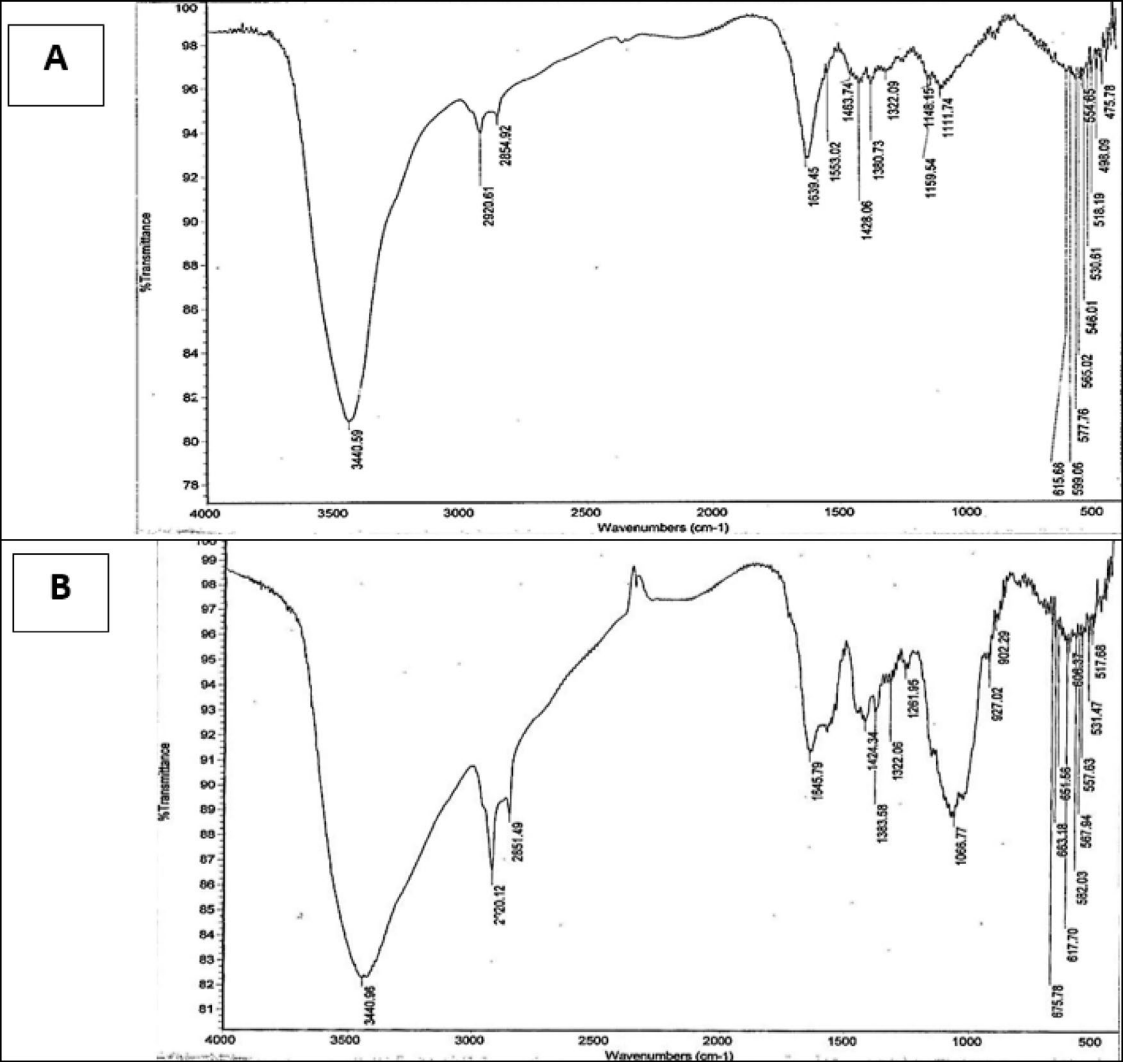
FTIR graphs of chitosan (**A**), and chitosan/EPS composite (**B**) showing several functional groups.

The biological materials exhibited various functional groups capable of adsorbing toxic dyes in aquatic environments. Exopolysaccharide used in the current study was produced from a potent EPS producing bacterial strain *Bacillus subtilis* EA5 (OR501464) which isolated from sugar cane juice^26^. Furthermore, the produced EPS was characterized using FTIR revealing a characteristic functional group including; hydroxyl (OH), amino (NH), methylene (CH_2_), carbonyl (C = O), phosphate (PO_4_) and C-N stretching groups in our published paper^26^. Fourier Transform Infrared Spectroscopy (FTIR) analysis of the EPS, chitosan, and the synthesized chitosan/EPS composite, conducted prior to the biosorption process, confirmed the presence of multiple functional groups (Table 1). These included hydroxyl (OH) at 3430, 3440 cm^− 1^, amino (NH) at 3440 and 1261 cm^− 1^, methylene (CH_2_) at 2934, 2920 cm^− 1^, and carbonyl (C = O) at 1639, 1645, 1647, and 1553 cm^− 1^. Additionally, phosphate (PO_4_) and C-N stretching groups were identified in the EPS and chitosan/EPS composite (Fig. 1).

**Table 1:** FTIR analysis of bacterial exopolysaccharide, chitosan and chitosan/EPS composite before MB biosorption.

EPS	Chitosan	Chitosan/EPS composite	Assignment
3430	3440.59	3440.96	Stretching and shifting of OH and N-H bands
2934.29	2920.61	2920.12	C–H stretching
-	2854.92	2851.49	
1647.77	1639.45	1645.79	C=O stretching vibration
-	1553.02	-	Shifting of C=O group
1455.09	1463.74	-	C–H bending
-	1428.06	1424.34	
1362.64	1380.73	1383.58	
-	1322.09	1322.06	
1269.15	-	1261.95	C–N stretching
-	1159.54	-	
-	1148.15	-	
1128.18	1111.74	-	
1068.85	-	1066.77	
1015.40	-	-	
926.75	-	927.02	PO4^−3^ stretching
878.38	-	902.29	

### Scanning electron microscopy of the biosorbents

**Fig. 2:**
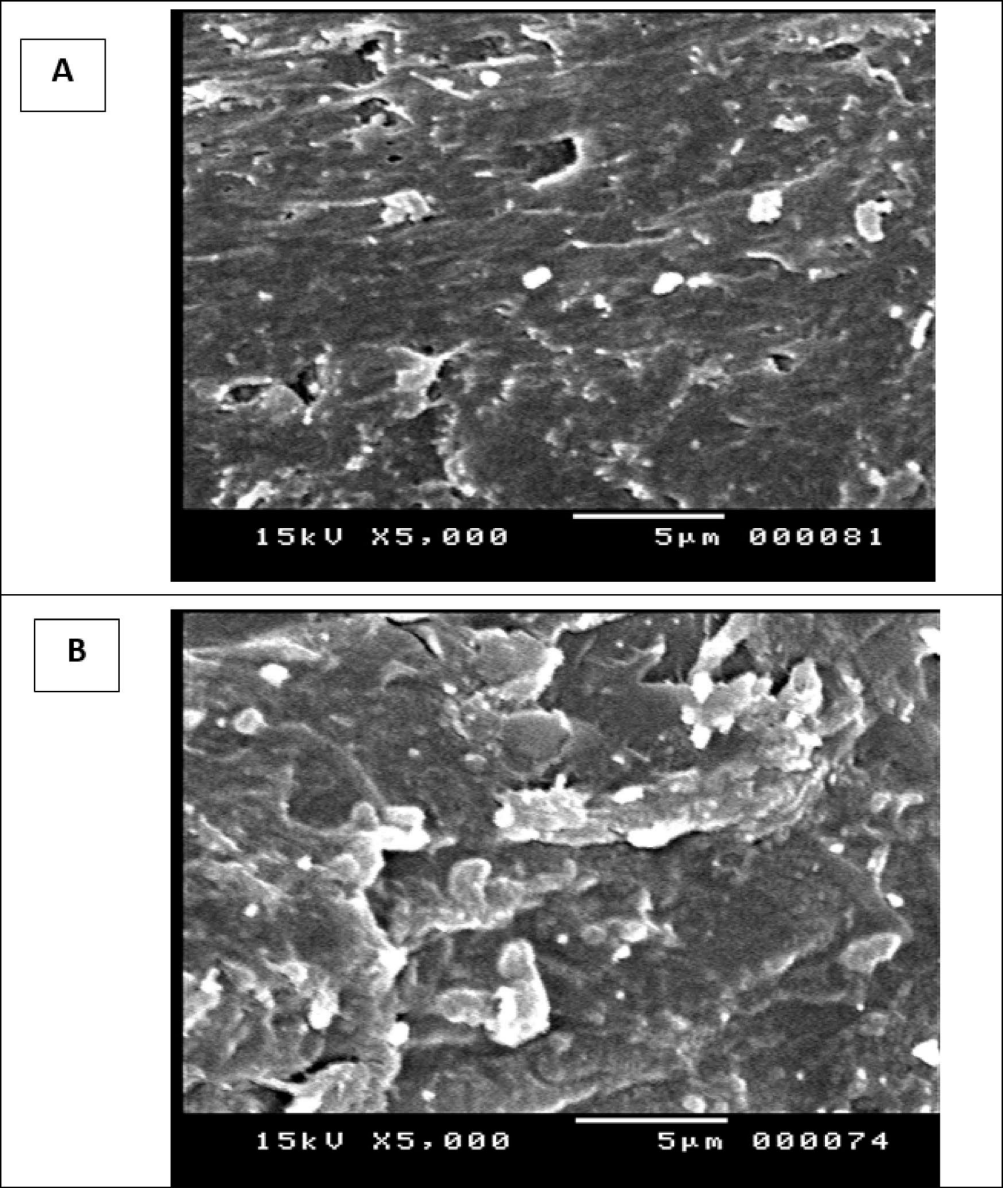
Scanning electron microscope graphs of chitosan (**A**), and chitosan/EPS composite (**B**).

The electron microscopic analysis showed a slightly uniform structure of chitosan with relatively smooth and homogeneous surface, indicating a dense structure with limited porosity. However, in the chitosan/EPS composite appeared more densely bound by a polysaccharide coating with a more irregular and porous surface with noticeable roughness and interconnected voids, as observed in (Fig. 2). The incorporation of EPS seems to disrupt the uniformity of chitosan, creating a heterogeneous structure that could enhance adsorption efficacy, improve mechanical flexibility, and provide better sites for functionalization or bioactivity.

### Impact of pH on removal of MB

The study examined how pH levels affected the biosorption of methylene blue (MB) by chitosan and the chitosan/EPS composite. The experiment was conducted at 30 °C using an initial MB concentration of 50 mg L⁻¹ and a contact time of 15 min.

**Fig. 3:**
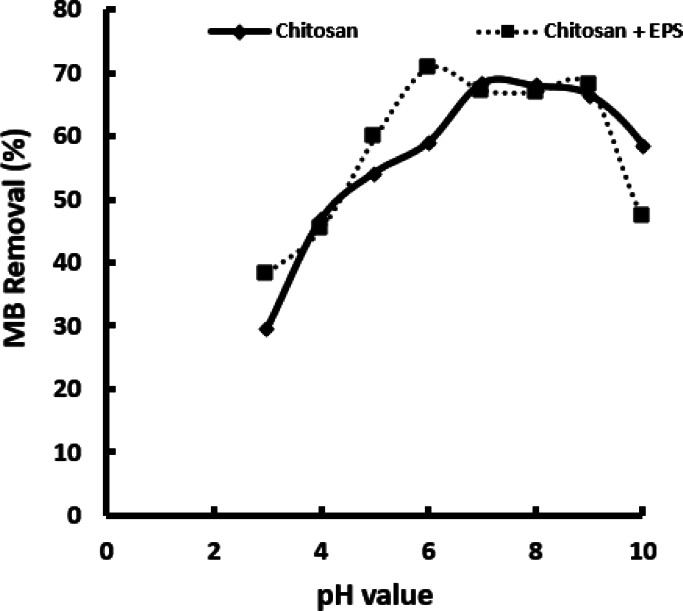
Impact of pH on MB decolorization.

The findings indicated that the level of MB decolorization rose as the pH of the solution increased from 3.0 to 7.0 for chitosan and from 3.0 to 8.0 for the chitosan/EPS composite. In the pH range of 6 to 9 for chitosan and 7 to 9 for the chitosan/EPS composite, there was no significant change in adsorption capacity, suggesting saturation of adsorption sites. Figure 2 indicated that MB adsorption on both chitosan and the chitosan/EPS composite was more effective at higher pH levels. Conversely, at lower pH values, the interaction between active sites and cationic MB molecules was hindered by proton competition, limiting adsorption efficiency^22^. Adsorption of MB by both materials was restricted at pH values below 4 due to electrostatic repulsion (Fig. 3). When the initial pH exceeded 4, both chitosan and the chitosan/EPS composite exhibited a predominantly negative charge, leading to strong electrostatic attraction to the MB dye. pH 6 was found to be optimal for MB adsorption using the chitosan/EPS composite, while pH 7 was optimal for chitosan, achieving removal efficiencies of 70.77 and 67.23%, respectively. Subsequent experiments were conducted under neutral conditions to investigate the adsorption of Methylene Blue (MB) by these adsorbents. Previous studies have highlighted the significant impact of pH on the adsorption capacity of polymers for MB. Changes in pH can affect the activity of surface functional groups on adsorbents and modify the chemical properties of dyes by influencing their ionization, which is regulated by the solution’s pH level^27^. Li et al.^27^ observed that the decreased adsorption efficiency at lower pH levels is due to the elevated H^+^ ion concentration. These ions compete with methylene blue (MB) for the same binding sites, reducing the negative charge on chitosan or the chitosan/EPS composite^28^. The difference in optimal pH values for chitosan (pH 7) and the chitosan/EPS composite (pH 6) is primarily related to the surface chemistry and the functional groups present on each adsorbent. The lower optimal pH for the composite (pH 6) compared to chitosan (pH 7) is advantageous, as it suggests effective performance under mildly acidic conditions commonly found in industrial wastewaters, reducing the need for stringent pH adjustment.

### Impact of contact duration

**Fig. 4:**
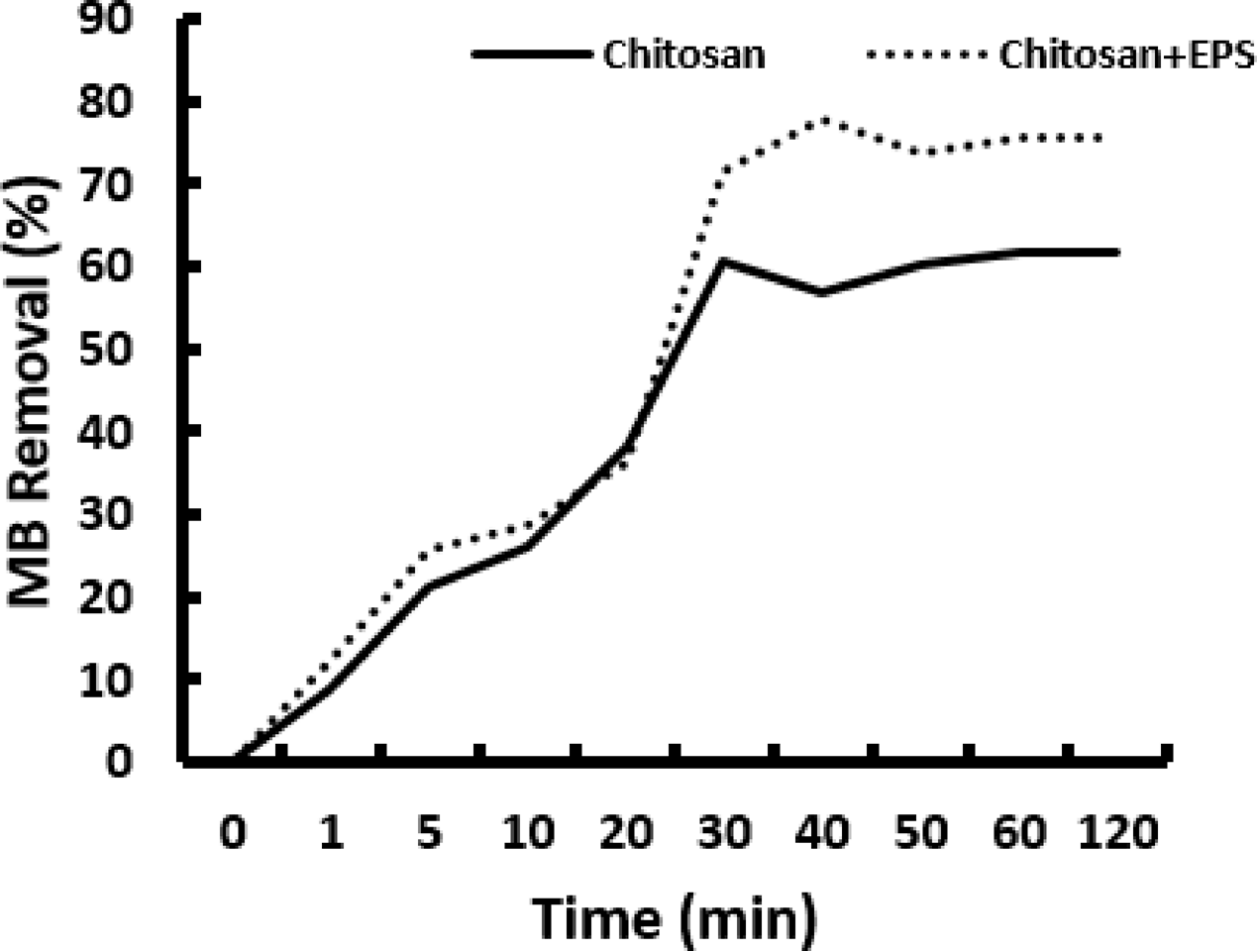
Effect of contact time on the removal of MB.

Figure 4 illustrates the impact of contact duration on the decolorization of methylene blue (MB) by chitosan and the chitosan/EPS composite. Both materials efficiently removed MB in approximately 30 min, with the composite achieving a decolorization rate of 71.6% compared to chitosan’s 60.62%. After 30 min, there was no significant improvement in removal efficiency, suggesting that equilibrium had been reached.

### Effect of initial dye concentration

**Fig. 5:**
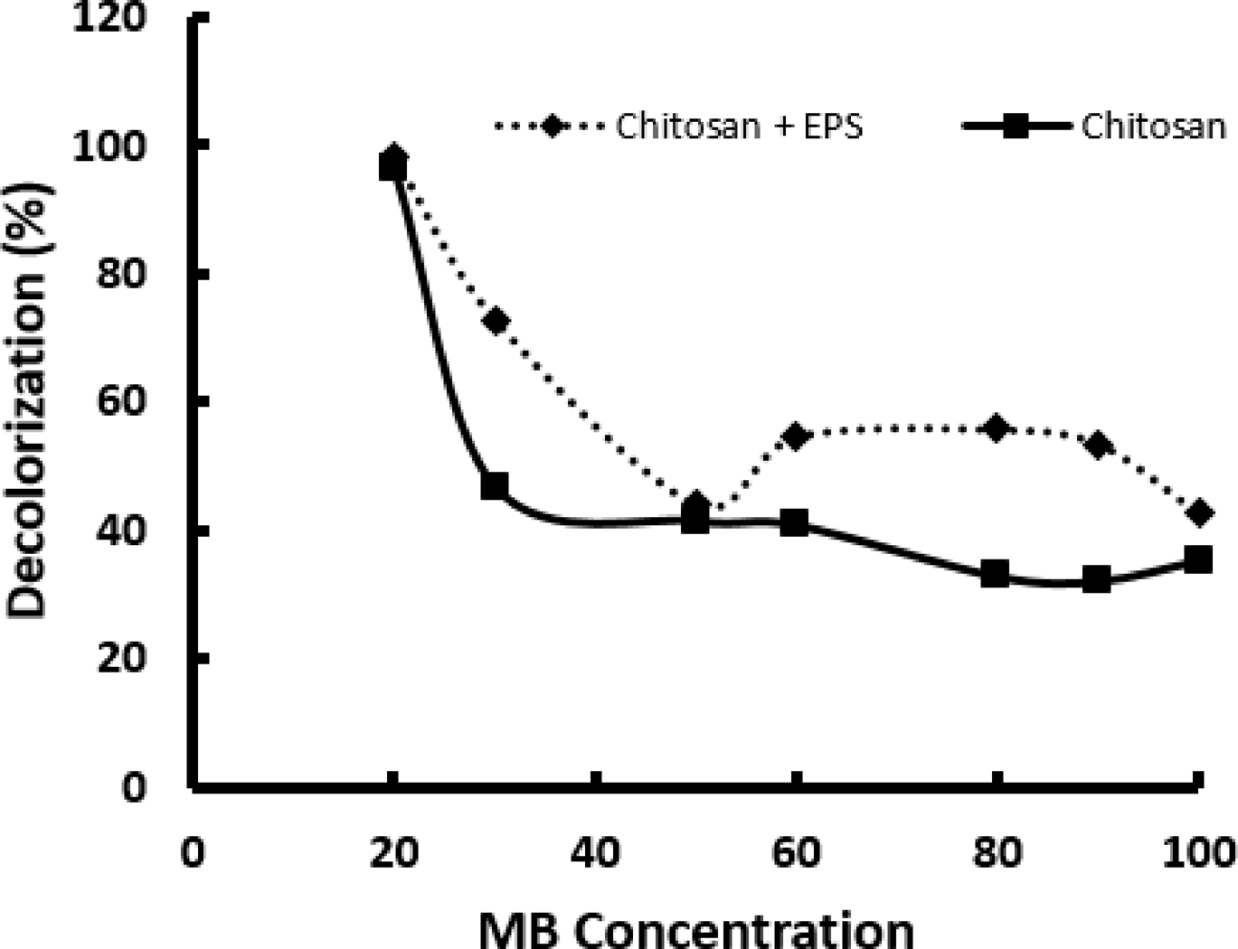
Effect of MB concentration on the removal efficiency.

The concentration of dyes in industrial wastewater can vary widely depending on the specific industry and methods used. It is crucial to understand the adsorption characteristics at different initial dye concentrations. Experimental results indicate that as the initial concentration of methylene blue (MB) increases, the dye removal efficiency of both chitosan and the chitosan/EPS composite decreases (Fig. 5). This trend has been observed in various studies^29–33^. Changes in initial MB concentrations during tests with chitosan and the chitosan/EPS composite show similar effects on adsorption efficiency: higher MB concentrations lead to improved removal rates. As the initial MB concentration increases, the transport rate of MB molecules to the adsorbent surface also increases^34^. However, the reduced removal efficiency at elevated MB concentrations may be due to a limited number of available active sites for adsorption. Consequently, at high MB concentrations, the saturation of these adsorption sites results in a decrease in removal efficiency^32^.

### Biosorption isotherm

The adsorption data for methylene blue using chitosan or the chitosan/EPS composite were evaluated using Langmuir and Freundlich isotherms. The Langmuir equation can be represented mathematically as follows:

1 $$\:{\mathrm{q}}_{\mathrm{e}\mathrm{q}}=\frac{{\mathrm{q}}_{\mathrm{m}\mathrm{a}\mathrm{x}}\times\:\mathrm{b}\times\:{\mathrm{C}}_{\mathrm{e}\mathrm{q}}}{1\:+\:\mathrm{b}\times\:{\mathrm{C}}_{\mathrm{e}\mathrm{q}}\:}$$

and the linear Langmuir formula is

2 $$\:\frac{{\mathrm{C}}_{\mathrm{e}\mathrm{q}}}{{\mathrm{q}}_{\mathrm{e}\mathrm{q}}}\:=\:\frac{1}{{\mathrm{q}}_{\mathrm{m}\mathrm{a}\mathrm{x}}\times\:\mathrm{b}}\:+\:\frac{{\mathrm{C}}_{\mathrm{e}\mathrm{q}}}{{\mathrm{q}}_{\mathrm{m}\mathrm{a}\mathrm{x}}}$$

C_eq_ = the equilibrium concentration of methylene blue (mg L^− 1^),

q_eq_ = the amount of adsorbed dye at equilibrium (mg g^− 1^),

q_max_ = the maximum biosorption capacity of the dye per gram of the adsorbent used (either chitosan or chitosan/EPS composite) (mg g^− 1^),

b = the Langmuir constant (L mg^− 1^), which indicates the affinity between the biosorbent and MB (L mg^− 1^).

**Fig. 6:**
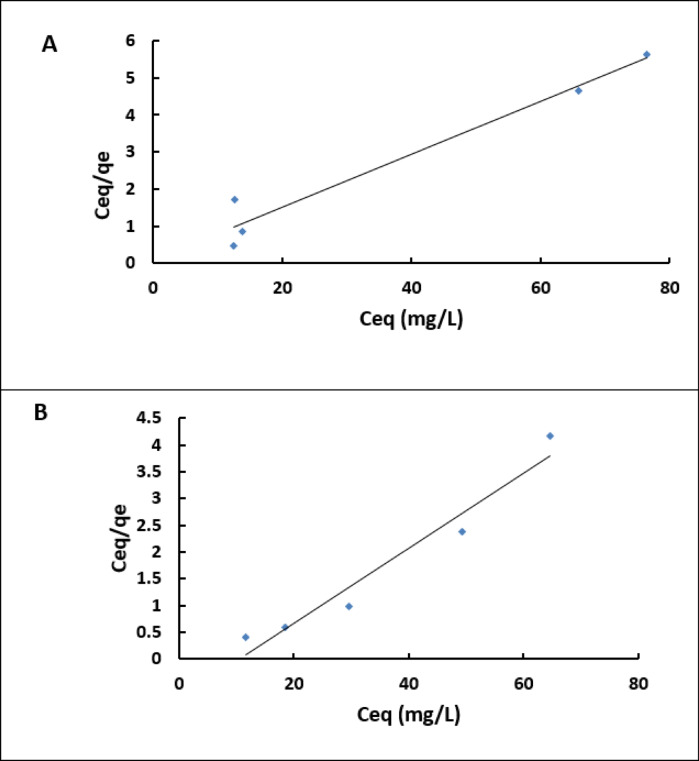
Shows the linear form of the Langmuir adsorption isotherm for the biosorption of Methylene blue by Chitosan/EPS composite (**A**) and Chitosan (**B**).

**Fig. 7:**
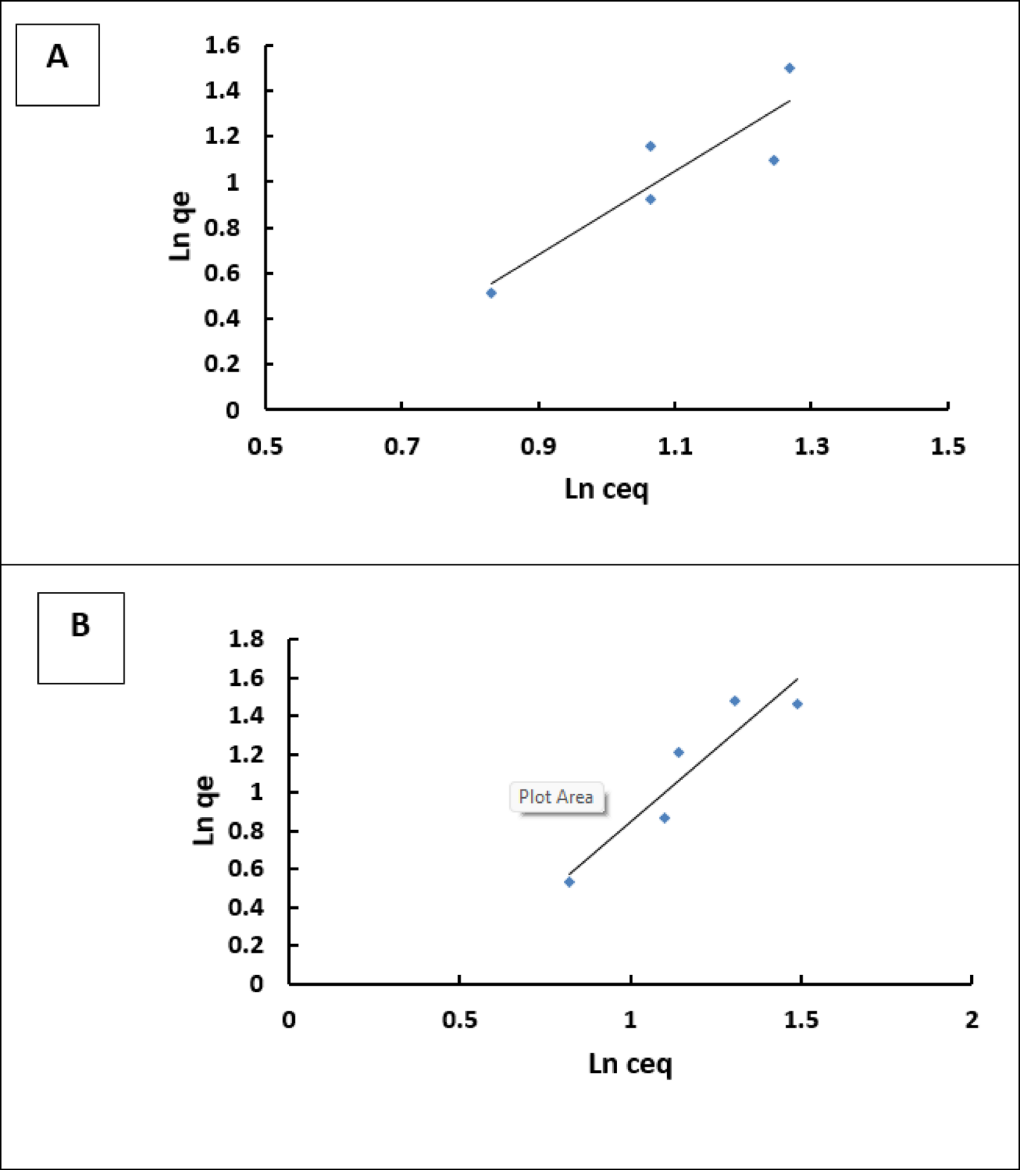
Shows the linear form of the Freundlich adsorption isotherm equation for Methylene blue by Chitosan/EPS composite (**A**) and Chitosan (**B**).

The values (b) and qmax can be calculated from the intercept and slope of the plot of C_eq_/q_e_ against the C_eq_ plot (Fig. 6). Adsorption equilibrium isotherms illustrate the relationship between the absorbed quantity of adsorbate and the remaining amount in the solution. The biosorption of methylene blue (MB) was studied using chitosan and the chitosan/EPS composite at initial concentrations ranging from 10 to 100 mg L^− 1^. The contact time was 30 min at pH levels of 6 and 7. The adsorption process follows the Langmuir isotherm, supported by high correlation coefficients (0.9489 for chitosan and 0.962 for the chitosan/EPS composite), indicating a homogeneous and monolayer adsorption mechanism for MB on both materials^35^. Comparable results have been reported for magnetic PS-EDTA resin^35^, and other polysaccharide hydrogels^36^. The Langmuir parameters, summarized in (Table 2), show that the maximum biosorption capacities (q_max_) for MB are 13.6986 mg g^− 1^ for chitosan and 14.2552 mg g^− 1^ for the chitosan/EPS composite. These results suggest that the chitosan/EPS composite has a higher biosorption capacity for MB compared to chitosan alone. The b values for chitosan and the chitosan/EPS composite in removing MB are 0.0993 and substantially higher 0.9743 L mg^− 1^ for the chitosan/EPS composite, demonstrating a much stronger affinity for MB molecules. The Langmuir constant (L mg^− 1^) is associated with the energy of adsorption. The Freundlich parameters KF [L mg^− 1^] and *n* refer to adsorption capacity and intensity, respectively^37^. The data best aligns with the Langmuir model, suggesting that adsorption takes place on a uniform monolayer surface of the adsorbents. A favorable adsorption process is suggested by the Langmuir and Freundlich models, as indicated by values of *n*< 1 (Table 2; Fig. 7). Wei et al.^17^ discovered that the Langmuir isotherm model provides a more accurate description of the adsorption of MB when utilizing exopolysaccharides obtained from aerobic granular sludge as adsorbents.

**Table 2:** The Langmuir and Freundlich adsorption isotherm constants for the biosorption of MB by chitosan and chitosan/EPS composite.

Biosorbents	Langmuir			Freundlich		
	q_max_ (mg g^− 1^)	b (L mg^− 1^)	R2	K_f_	*n*	R2
Chitosan	13.6986	0.0993	0.9489	9.278	0.5467	0.8004
Chitosan/EPS composite	14.2552	0.97431	0.962	1.1715	0.65167	0.8747

### Biosorption kinetics

The kinetic data from the biosorption experiments were analyzed using pseudo-first order (PFO) and pseudo-second order (PSO) models, as described by Ho and McKay^38^. The pseudo-first order model is given by the following equation:

3 $$\:Log\:\left(qe\:-\:qt\right)=log\:qe-\left(\frac{\mathrm{k}1.\mathrm{a}\mathrm{d}\mathrm{s}}{2.303}\right)t$$

In the equation, *q*_*e*_ and *q*_*t*_ (in mg/g) denote the quantity of methylene blue (MB) adsorbed at equilibrium and at time *t* (in minutes), respectively, while *k*1 is the rate constant (min⁻¹).

**Fig. 8:**
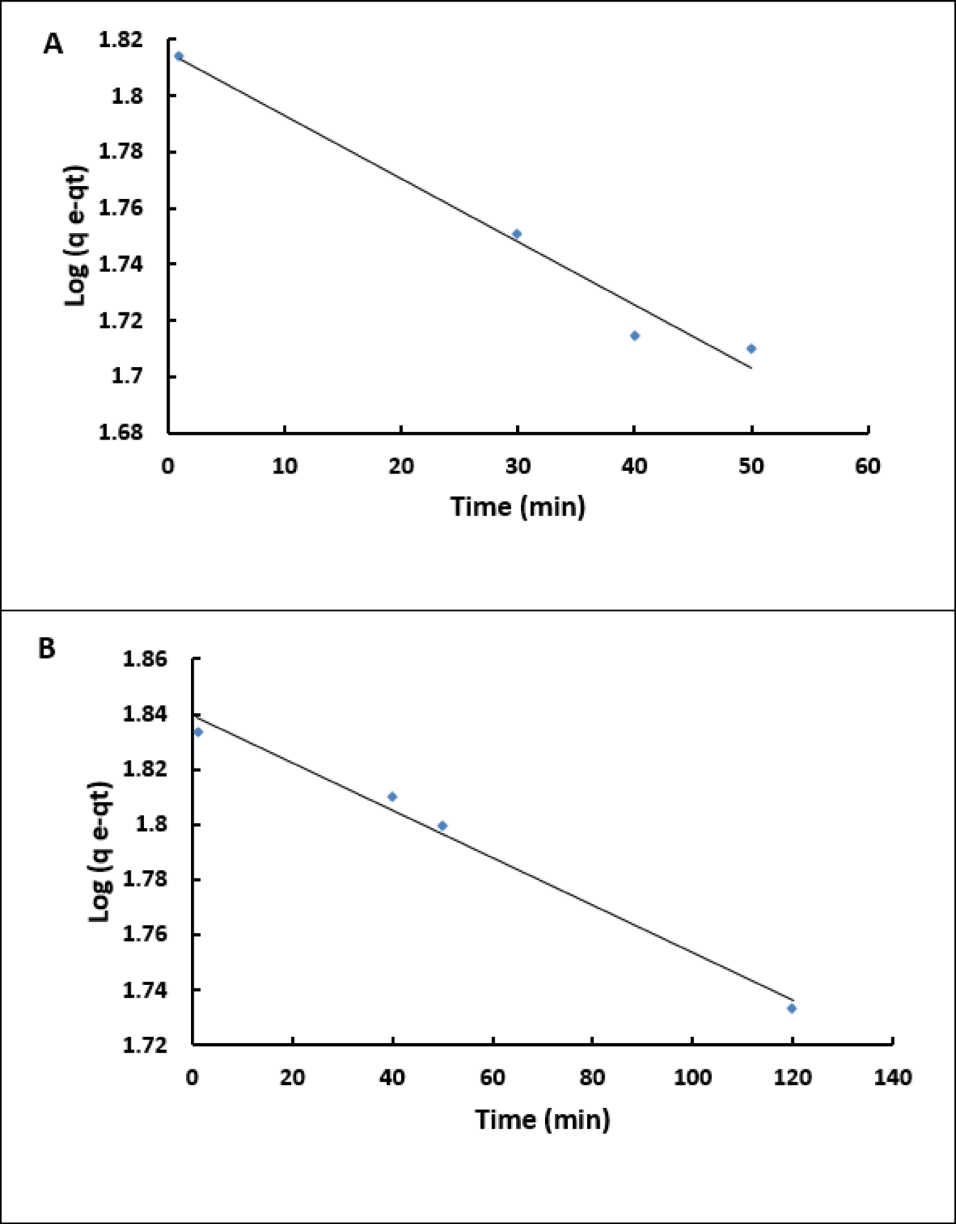
The pseudo-first-order kinetics plots of MB adsorption on Chitosan (**A**) and chitosan/EPS composite (**B**).

**Fig. 9:**
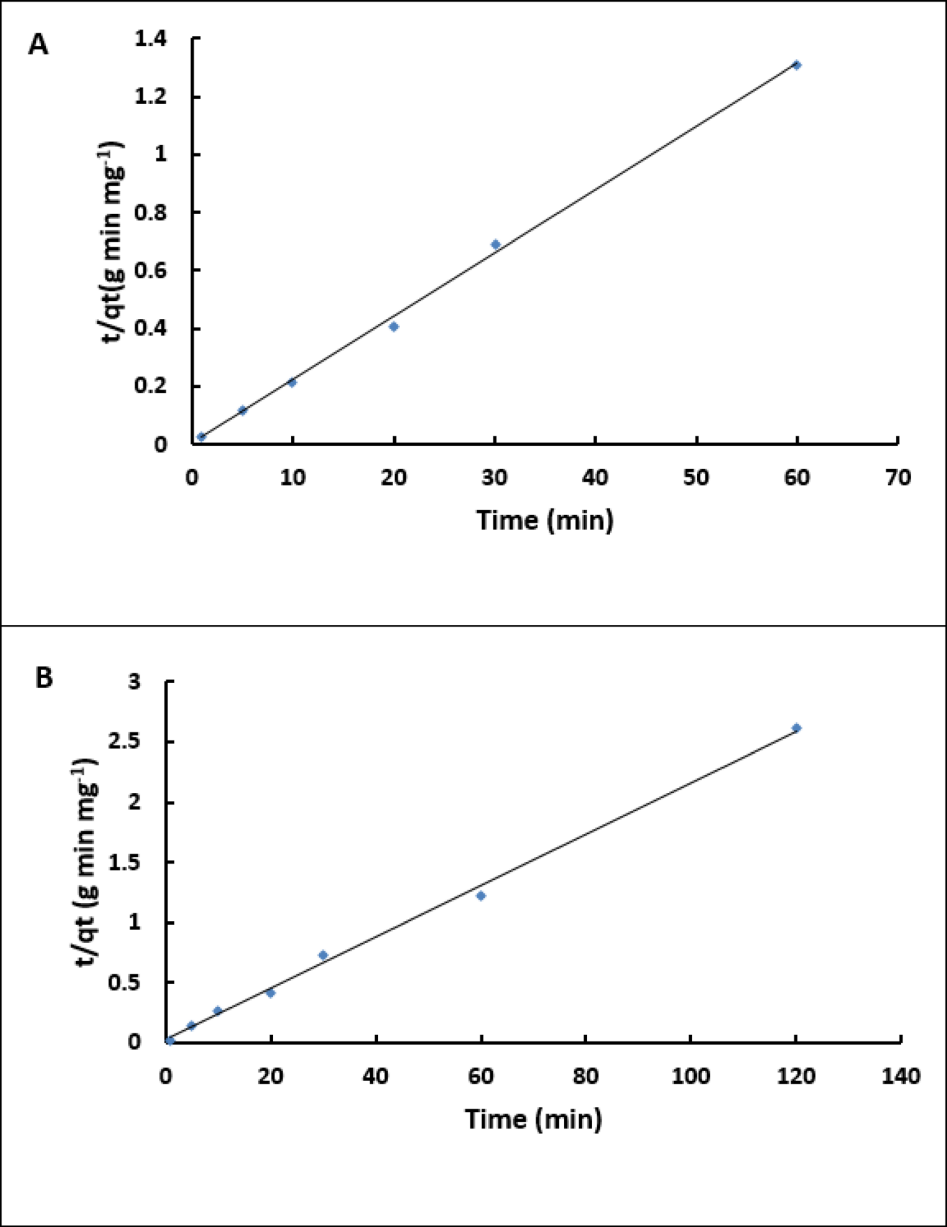
The pseudo-second-order kinetics plots of MB adsorption on chitosan (**A**) and chitosan/EPS comoposite (**B**).

If the calculated *q*_*e*_ does not match the equilibrium metal uptake, it indicates that first-order kinetics do not apply to the reaction. The linear plots of log (*q*_*e*_−*q*_*t*_) versus *t* for the pseudo-first-order model related to MB adsorption by chitosan and the chitosan/EPS composite are illustrated in (Fig. 8). Figures 8 and 9 display the fitting plots for the PFO and PSO models, demonstrating a good fit of the kinetic data, as evidenced by the relatively high *R*^2^ values provided in (Table 3).

The PFO model’s calculated *q*_*eq*_ (cal) values were notably lower than the experimental *q*_*e*_ values for the tested initial MB concentrations, suggesting that the PFO model is not a suitable descriptor of the adsorption kinetics. The correlation coefficients for the pseudo-first order equations for chitosan and the chitosan/EPS composite were 0.977 and 0.989, respectively. The calculated *q*_*eq*_ values did not match the experimental *q*_*e*_ values, suggesting that the pseudo-first order model is not suitable for these biosorption experiments (Table 3). To elucidate the sorption kinetics more effectively, the study utilized a pseudo-second order model as proposed by Ho and McKay^38^.

This model and its linear form were described in previous researches^1,39–42^. The linear plots of the pseudo-second order model for MB adsorption by chitosan and the chitosan/EPS composite are shown in (Fig. 9). Table 3 presents the rate constants and correlation coefficients for the adsorption of MB. The correlation coefficient for the pseudo-second order adsorption was determined to be 0.998 for chitosan at an initial concentration of 50 mg L^− 1^, and 0.9973 for the chitosan/EPS composite. The biosorption capacities calculated using the pseudo-second order model were 45.87 mg g^− 1^ for the chitosan/EPS composite and 41.67 mg g^− 1^ for chitosan, closely matching the experimental values. Notably, the rate constant (K₂) for the composite (0.176 g mg^− 1^ min^− 1^) was an order of magnitude higher than that of chitosan (0.0023 g mg^− 1^ min^− 1^), unequivocally confirming the faster adsorption kinetics achieved by the composite material. This suggests that the pseudo-second order model is better suited for explaining the biosorption kinetics of MB by both chitosan and the chitosan/EPS composite. Comparable adsorption patterns have been noted in altered, activated carbon, chitosan beads, chitosan hydrogels and alginate-like exopolysaccharide^1,39,40,43^.

**Table 3:** Kinetic parameters derived from the pseudo-first-order and pseudo-second-order models for the biosorption of MB.

Adsorbent	Initial conc. (ppm)	q_exp_ (mg g^− 1^)	Pseudo first order			Pseudo second order		
			K_1_	q_e_	*R*_2_	K_2_	q_e_	*R*_2_
Chitosan	50	43.23	0.004	6.534	0.97747	0.0023	41.67	0.9973
Chitosan/EPS composite	50	41.53	0.0207	6.911	0.98882	0.176	45.87	0.9981

### FTIR analysis after MB biosorption

**Fig. 10:**
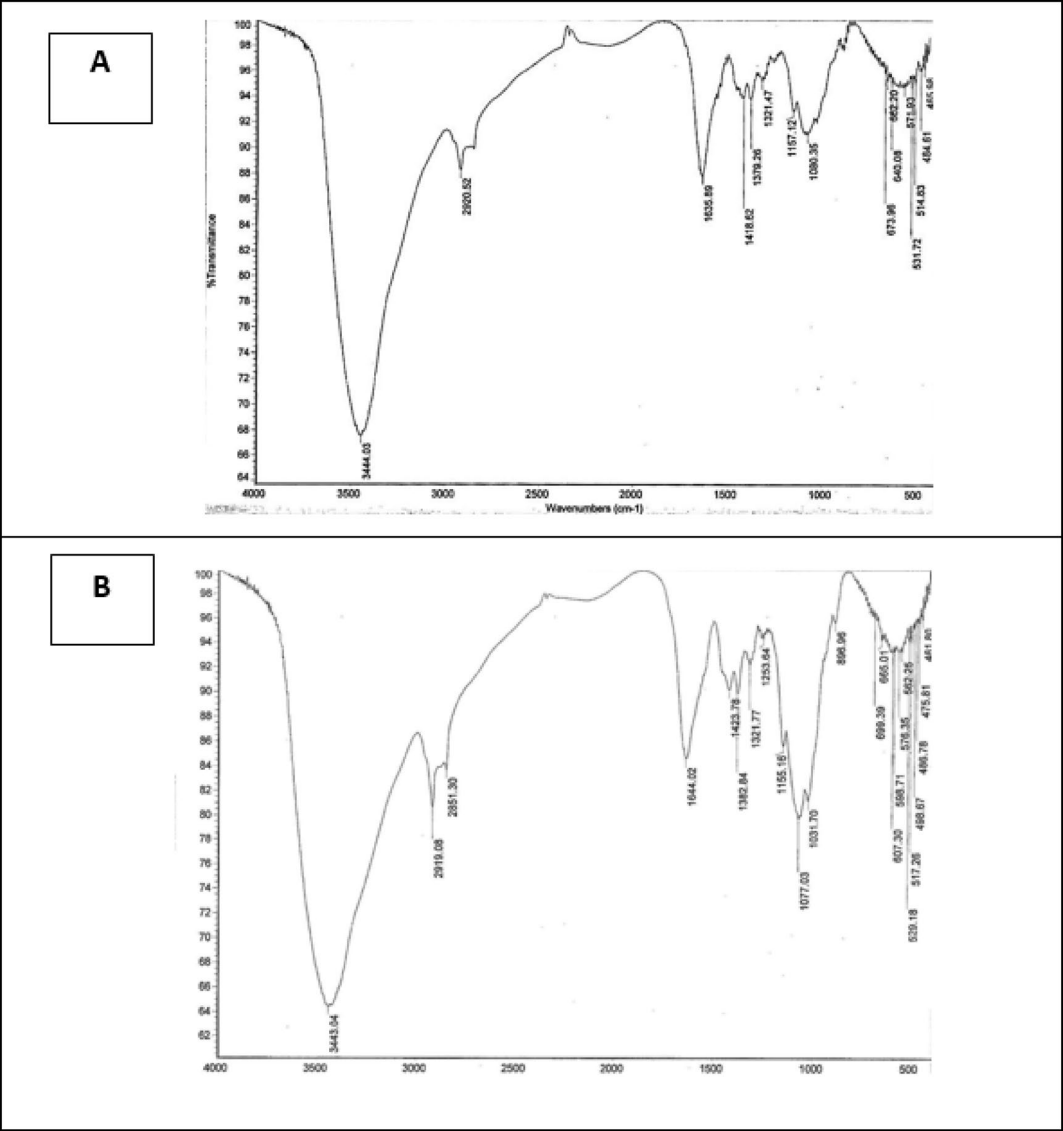
FTIR graphs of chitosan (**A**) and chitosan/EPS composite (**B**) showing several functional groups.

FTIR analysis after methylene blue (MB) biosorption revealed notable shifts in chitosan peaks from 3440, 1639, 1428, 1380, and 1959 cm⁻¹ to 3444, 1635, 1417, 1379, and 1957 cm⁻¹, respectively (Fig. 10A; Table 4). The chitosan/EPS composite exhibited similar changes, with initial peaks at 3440, 2920, 1645, 1424, 1383, 1322, 1261, 1066, and 902 cm⁻¹ shifting to 3443, 2919, 1644, 1423, 1382, 1321, 1155, 1077, and 896 cm⁻¹, respectively (Fig. 9B; Table 4). These spectral variations confirm the involvement of functional groups such as OH, NH, C = O, PO₄³⁻, and C–N in MB binding.

Li et al.^41^ reported that the monosaccharide composition and molar ratios in EPS-R040 influence its morphology and adsorption performance. Their study demonstrated hydrogen bonding between MB’s nitrogen and the hydroxyl hydrogen of EPS-R040, facilitating dye–polymer interactions. These mechanistic insights are consistent with kinetic modeling, where the pseudo second order model confirms chemisorption as the dominant pathway^44,45^. The FTIR findings provide direct spectroscopic evidence for the proposed adsorption mechanisms, which is consistent with the kinetic and isotherm modeling, collectively explaining the enhanced performance of the chitosan/EPS composite. The synergistic integration of chitosan and EPS provides abundant active sites and amplifies electrostatic attraction, resulting in enhanced adsorption capacity and faster equilibrium compared to chitosan alone.

**Table 4:** FTIR analysis of Chitosan and Chitosan/EPS composite functional groups after MB biosorption.

Wave numbers (Cm^− 1^)		Assignment
Chitosan	chitosan/EPS composite	
3444.03	3443.04	Stretching and shifting of OH and N-H bands
2920.50	2919.08	C-H stretching
-	2851.30	C-H stretching
1635.89	1644.02	C = O stretching vibration
1418.62	1423.78	Shifting of C = O group
1379.26	1382.84	C-N stretching
1321.47	1321.77	
-	1253.64	
1157.12	1155.16	
-	1077.03	
-	1031.70	
1080.35	896.96	

**Fig. 11:**
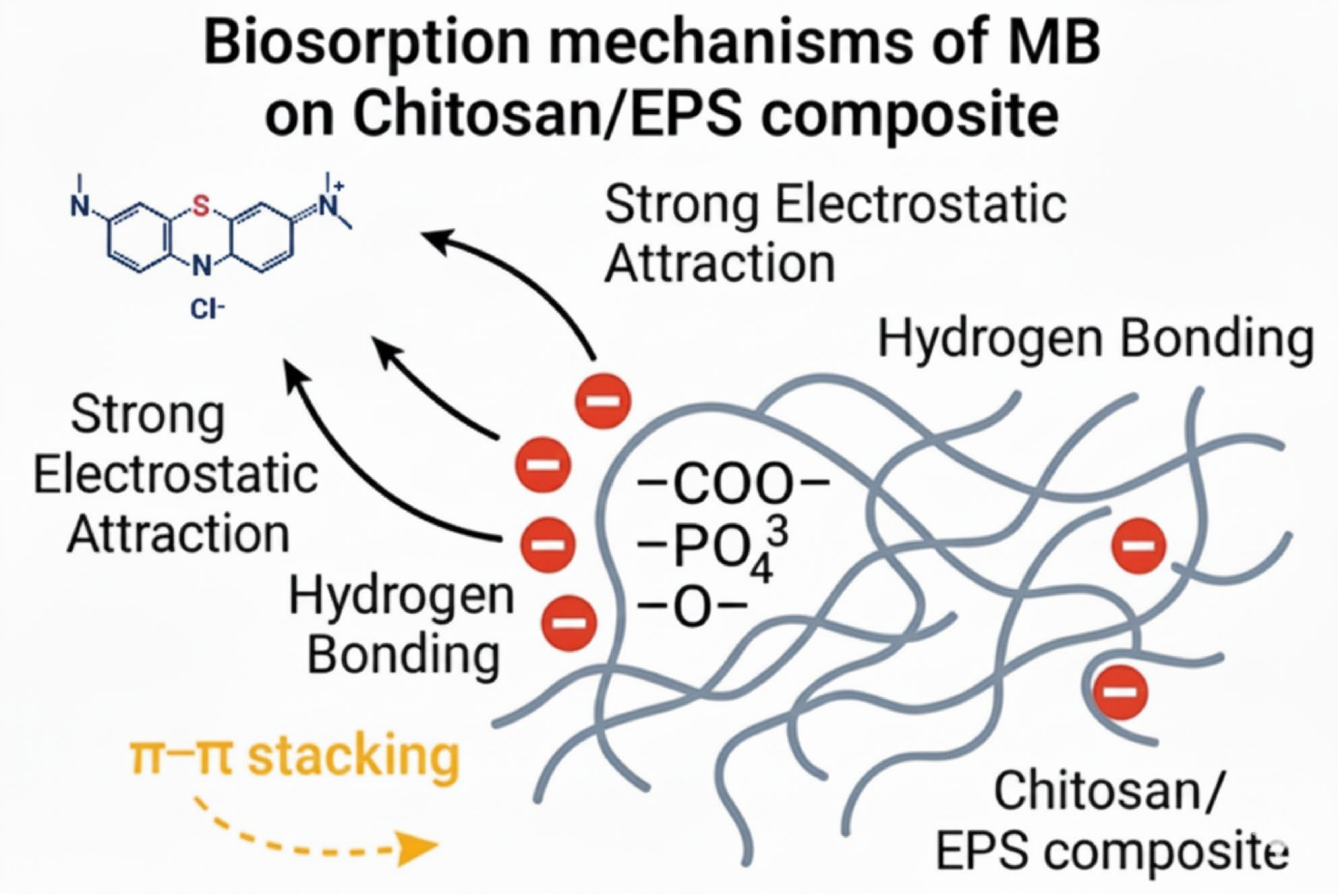
Schematic representation of methylene blue (MB⁺) biosorption by a chitosan/EPS composite.

The present work demonstrates clear synergistic improvement when chitosan is combined with *Bacillus subtilis* exopolysaccharide (EPS). The composite achieved a maximum monolayer adsorption capacity of 14.26 mg g⁻¹ (vs. 13.70 mg g⁻¹ for chitosan), a 71.6% removal efficiency (vs. 60.6% for chitosan only), and an order-of-magnitude higher pseudo-second-order rate constant (K₂ = 0.176 g mg⁻¹ min⁻¹ vs. 0.0023 g mg⁻¹ min⁻¹), reaching equilibrium in less than 30 min. These gains arise from the dramatically increased density of anionic binding sites (carboxylate, phosphate, and deprotonated hydroxyl groups) introduced by EPS, as evidenced by pH-edge experiments and post-adsorption FTIR shifts. The dominant biosorption mechanisms as shown in (Fig. 11), deduced from pH dependence, spectroscopic evidence, and kinetic modeling, are (i) strong electrostatic attraction between the cationic dimethylamino groups of MB and the negatively charged –COO⁻, –PO₄³⁻, and –O⁻ sites on the composite surface^46^; (ii) extensive hydrogen bonding between MB heteroatoms (N, S) and polysaccharide hydroxyl/carboxyl groups; and (iii) π–π stacking interactions between the aromatic rings of MB and the polysaccharide backbone^46^. The incorporation of EPS not only multiplies available anionic groups but also creates a more hydrophilic, swollen hydrogel network that facilitates rapid dye diffusion—explaining both the enhanced capacity and markedly faster kinetics compared to chitosan alone. Beyond performance, the composite offers significant environmental and economic advantages: EPS is valorized from a low-value fermentation by-product into a functional material, fully aligning with circular-economy principles. The preparation protocol is simple, scalable, and free of toxic cross-linkers (e.g., glutaraldehyde or epichlorohydrin) commonly required in many chitosan-based adsorbents.

A comparative evaluation with recent literature (Table 5) confirms the competitiveness of the chitosan/EPS composite. It outperforms most unmodified natural biosorbents (e.g., alginate, raw activated carbon, and numerous agricultural wastes) in both adsorption capacity and equilibration time. Although some chemically modified or enzyme-blended chitosan materials exhibit slightly higher qₘₐₓ values (≈ 19–50 mg g⁻¹), they typically require several hours to reach equilibrium and involve complex, costly synthetic routes. In contrast, the present composite achieves > 70% removal in 30 min at near-neutral pH using only renewable, non-toxic biopolymers—characteristics highly desirable for integration into continuous-flow industrial systems. In practical terms, the chitosan/EPS composite represents a sustainable, low-cost, and rapidly acting biosorbent ideally suited for treating cationic-dye-laden effluents from the textile, leather, paper, and cosmetic industries. Its excellent performance under mild conditions, ease of preparation, and ability to valorize microbial waste position it as a promising next-generation green material for real-world dye wastewater remediation.

**Table 5:** Comparison of methylene blue biosorption performance between present study and selected reported biosorbents.

Biosorbent Material	q_max_ (mg g^− 1^)	Equilibrium Time (min)	Optimal pH	Reference
Chitosan–pectinase blend	16.81	30	7	^47^
Magnetic chitosan/organic rectorite	24.69	60	6	^48^
Activated lignin-chitosan pellets	36.25	-	7	^49^
Graphene oxide/chitosan/Fe_3_ O_4_	30.10	-	10.5	^50^
Chitosan/Activated Carbon	22.52	60	6.5	^46^
**Chitosan/EPS composite (present study)**	**14.26**	**30 min**	**6**	**This work**
**Chitosan (present study)**	**13.70**	**30 min**	**7**	**This work**

## Materials and methods

### Preparation of biosorbent

#### Exopolysaccharides (EPS) Preparation

A 10 mL suspension of *Bacillus subtilis* ES (OR501464) was inoculated into 500 mL Erlenmeyer flasks containing a sterile growth medium contained per liter: 0.2 g KH_2_PO_4_, 1.5 g K_2_HPO_4_, 0.2 g Na_2_SO_4_, 0.002 g FeCl_3_, 1 g NaNO_3_, 40 g sucrose, with a pH adjusted to 9. The flasks were incubated at 30 ± 2 °C for 48 h. Post-incubation, the culture broth was centrifuged at 6000 rpm for 15 min to separate the bacterial cells from the supernatant. The supernatant was then collected, and cold ethanol (twice the volume of the supernatant) was added to precipitate the EPS. The mixture was gently stirred and left overnight at 4 °C. Subsequently, it was centrifuged at 10,000 rpm for 10 min at 4 °C to obtain the bacterial EPS precipitate. The collected EPS was washed with cold ethanol to remove impurities and then dried until a constant weight was achieved.

#### Chitosan

Chitosan was sourced from the Chemistry Department at the Faculty of Science, Assiut University, with a molecular weight of 360.8 kDa and a degree of deacetylation ≥ 85%.

### Preparation of chitosan/exopolysaccharide composite

Chitosan (1 g) was dissolved in a 100 mL solution containing 1% acetic acid by stirring continuously until completely dissolved. Subsequently, 1 g of EPS was added and thoroughly mixed to achieve uniformity. The pH was then adjusted to 7, and the mixture was heated to 70 °C for 3 h to promote composite formation. The gel-like composite obtained was cooled, centrifuged to separate the composite, dried overnight at 45 °C, and stored at 4 °C.

#### Characterization of the biosorbent

FTIR analysis was conducted on the composite to determine the functional groups that are involved in the biosorption of dye pollutants. The surface morphologies of the chitosan and chitosan/EPS composite were observed with a scanning electron microscope. SEM analysis was performed using a JEOL JSM-5400 L.V. SEM instrument at Assiut University, Egypt, to study the surface morphology of the biosprpents.

### Adsorption experiments

#### Effect of contact time on methylene blue (MB) removal

Fifty milligrams of the prepared composite or chitosan alone were mixed with 50 mL of a 50 mg L⁻¹ methylene blue (MB) solution. The mixtures were stirred at 120 rpm and maintained at 28 ± 2 °C. The initial solution pH was adjusted to 7.0. Samples were taken at specified time intervals (0–60 min), centrifuged at 8,000 rpm for 5 min. The absorbance at 663 nm was measured using a spectrophotometer to determine the concentration of MB in the supernatant.

#### Impact of pH on the removal of methylene blue (MB)

The impact of pH on methylene blue (MB) removal was investigated by adding 50 milligrams of the developed composite or chitosan alone to a 50 mL solution containing 50 mg L⁻¹ of methylene blue (MB). The solution’s pH was adjusted from 1.0 to 7.0. The mixtures were stirred at 120 rpm and maintained at 28 ± 2 °C. Samples were taken at various time points from 0 to 60 min. After incubation, the mixture was centrifuged, and the remaining dye concentration was measured at 663 nm.

#### Biosorption isotherms of methylene blue

The biosorption isotherms of methylene blue (MB) were studied using the Langmuir and Freundlich models to characterize the equilibrium relationship between the adsorbate and the adsorbent. In the isotherm experiments, 20 mg of the biosorbent (either the composite or chitosan alone) was mixed with varying concentrations of MB in 20 mL of deionized water at pH 6.0. The mixtures were agitated at 120 rpm for 30 min at 28 ± 2 °C. following agitation, the mixtures were spun at 8,000 rpm for 5 min, and the resulting supernatant was analyzed using a spectrophotometer to measure the remaining dye concentration.

### Methylene blue (MB) removal

The biosorption of Methylene Blue was calculated using the following equations:

4 $$\:Methylene\:blue\:remaval\:\left(\%\right)=\frac{\left(C0-Ci\right)}{C0}\:x\:100$$

Where C0 is the initial dye concentration and Ci is the equilibrium dye concentration.

5 $$\:The\:amount\:of\:MB\:adsorbed\:\left(qt\right)=\:\:\:\frac{\left(C0-Ci\right)\:V}{W}\:$$

Where C0 is the initial dye concentration, Ci is the residual dye concentration, V is the solution volume (L), and W is the dry weight of the adsorbent.

Adsorption kinetics were evaluated using pseudo-first-order (PFO) and pseudo-second-order (PSO) kinetics.

### Analysis using fourier-transform infrared spectroscopy (FTIR)

The functional groups of the adsorbent were analyzed prior to and following the biosorption process using a FTIR spectrometer (Thermo Scientific Nicolet iS10 FT-IR Spectrometer, USA) with the KBr pressed disk procedure.

## Conclusion

This study demonstrates the effectiveness of a chitosan and *Bacillus subtilis* exopolysaccharides (EPS) composite in biosorbing methylene blue (MB). The optimal pH for dye removal was found to be 6 for the composite and 7 for chitosan, with significant decolorization achieved within approximately 30 min. The adsorption process followed the Langmuir isotherm model and pseudo-second order kinetics, indicating efficient monolayer adsorption. FTIR analysis post-adsorption confirmed interactions between MB and the biosorbents. These results suggest that chitosan/EPS composites show promise for wastewater treatment, offering potential for improved dye removal under optimized conditions. The simplicity of composite preparation, reliance on renewable biopolymers, and valorization of microbial EPS suggest that this approach could be scaled up cost-effectively for wastewater treatment. The process avoids harsh chemicals and energy-intensive steps, making it suitable for industrial applications. Furthermore, the rapid adsorption kinetics and high removal efficiency observed in this study indicate potential for integration into continuous flow systems, reducing treatment time and operational costs.

## Data Availability

The datasets of the nucleotide sequence of bacterial isolate *Bacillus subtilis* EA5 generated during the current study are available in the GenBank nucleotide sequence database under accession number (OR501464), the NCBI website: [https://www.ncbi.nlm.nih.gov/nuccore/OR501464.1/](https:/www.ncbi.nlm.nih.gov/nuccore/OR501464.1) .
